# Genome-wide identification, structural analysis and expression profiles of short internodes related sequence gene family in quinoa

**DOI:** 10.3389/fgene.2022.961925

**Published:** 2022-08-22

**Authors:** Xiaolin Zhu, Baoqiang Wang, Xian Wang, Xiaohong Wei

**Affiliations:** ^1^ College of Agronomy, Gansu Agricultural University, Lanzhou, China; ^2^ Gansu Provincial Key Laboratory of Aridland Crop Science, Gansu Agricultural University, Lanzhou, China; ^3^ College of Life Science and Technology, Gansu Agricultural University, Lanzhou, China

**Keywords:** quinoa, SRS gene family, evolutionary analysis, expression pattern, genome-wide analysis

## Abstract

Based on the whole genome data information of *Chenopodium quinoa Willd*, the CqSRS gene family members were systematically identified and analyzed by bioinformatics methods, and the responses of CqSRS genes to NaCl (100 mmol/L), salicylic acid (200 umol/L) and low temperature (4°C) were detected by qRT-PCR. The results showed that a total of 10 SHI related sequence genes were identified in quinoa, and they were distributed on 9 chromosomes, and there were four pairs of duplicated genes. The number of amino acids encoded ranged from 143 aa to 370 aa, and the isoelectric point ranged from 4.81 to 8.90. The secondary structure was mainly composed of random coil (Cc). Most of the SRS gene encoding proteins were located in the cytoplasm (5 CqSRS). Phylogenetic analysis showed that the CqSRS genes were divided into three groups, and the gene structure showed that the number of exons of CqSRS was between two-five. Promoter analysis revealed that there are a total of 44 elements related to plant hormone response elements, light response elements, stress response elements and tissue-specific expression in the upstream regin of the gene. Protein interaction showed that all 10 CqSRS proteins appeared in the known protein interaction network diagram in Arabidopsis. Expression profile analysis showed that CqSRS genes had different expression patterns, and some genes had tissue-specific expression. qRT-PCR showed that all SRS family genes responded to ABA、NaCl、drought and low-temperature treatments, but the expression levels of different CqSRS genes were significantly different under various stresses. This study lays a foundation for further analyzed the function of CqSRS genes.

## 1 Introduction

In the process of plant growth and development, plants will encounter a variety of abiotic stresses (drought, salt, low temperature, high temperature), as well as biological stresses such as bacteria and fungi, which have a great impact on plant yield and quality. Thus, to adapt to extreme environments, plants change at the genome-wide level (gene expression) to resist various stresses. Transcription factors (TFs) are critical to this process and can specifically bind to the promoter of target genes. The structure and function of TFs play a key role in regulating plant resistance to biotic and abiotic stresses (Boyer, 1982). SHI related sequence (SRS) gene family, also known as short internodes (SHI) or SHI/STY/family of SRS (for short internodes, stylish, and SHI-related sequence), is unique in plants. The family encodes a specific transcription factor with two different conserved areas, and the prediction proteins show the sequence of particularly high consistency in two areas, the first area is located in the protein amino acid sequence of N-terminal. The results show that the supposed ring domain contains 31 amino acid residues in the consistent sequence of Cys-X2-Cys-X7-Cys-X-His-X2-Cys-X2-Cys-X7-Cys-X2-X2-His. The motif is a C3HC3H ring domain and the domain is conservative ring zinc finger. The second conserved domain is located at the C-terminal, which is the only domain of proteins in the SHI family. This domain has four highly conserved residues, so it is named IGGH. In addition to these two conserved domains, the remaining protein sequences are highly differentiated (Fridborg et al., 1999), and these characteristics are particularly critical for their transcription factor functions. The SRS genes of *Arabidopsis thaliana* contain two conserved domains, zinc finger domain and IGGH domain, but the sequence is also highly differentiated. *SHI* is the first member of the SRS gene family and has been identified in *Arabidopsis* dwarf mutant SHI. This gene can inhibit GA response at the GA biosynthetic site, and the SRS/STY protein contains acidic amino acids, which is a characteristic of this family of proteins as transcriptional activators (Fridborg et al., 2001).

At present, a total of 11 SRS genes have been identified in maize (He et al., 2020), and 11 SRS genes in *Arabidopsis*, including *SHI*, *STY1*, *STY2*, *LRP1* and *SRS3*-*SRS8* (Kuusk et al., 2006; Sohlberg et al., 2006). Many SRS genes play important roles in regulating plant hormone biosynthesis, photomorphogenesis, metabolization-related material structure, signal transduction, and plant organ growth and development. For example, during the development of lateral roots (LR), *LRP1* is regulated by the auxin signal transduction mechanism (Ive et al., 2008; Bert et al., 2012), and auxin and histone deacetylation affect the expression of *LRP1*, and by regulating the dynamic balance of auxin in *Arabidopsis thaliana*. Meanwhile, it negatively regulates the development of *LRP* in the downstream of the auxin reaction module of LR, and it plays a role in the downstream of rootless and undetectable meristematic tissue 1 (*RUM1*), *RUM1* is an Aux/IAA protein that regulates the crown root development of corn (Zhang et al., 2015). *STY1*, *STY2* and *STY3* in *Lotus Japonicus*, as direct LiNF-YA1 targets, are involved in the formation of nodules (Hossain et al., 2016). *STY1* up-regulates auxin biosynthesis (Klund et al., 2010). Recent studies have shown that *Arabidopsis SRS5* gene is a positive regulator of photomorphogenesis, which can directly bind to promoters of photomorphogenesis genes (such as *HY5*, *BBX21* and *BBX22*) to activate its expression to promote photomorphogenesis. Meanwhile, *SRS5* is also a target of COP1-mediated degradation (Yuan et al., 2018). The *SHI*/*STY*/*SRS* genes play a conservative role in the apex of *Arabidopsis* regulatory network, and these genes guide the development of styles and stigmas (Gomariz-Fernández et al., 2017). Studies in rice showed that *OsSHI1* inhibited the transcriptional activity of *IPA1* and regulated plant structure by affecting the DNA binding activity of *IPA1* on the promoter region of *OsTB1* and *OsDEP1* (Duan et al., 2019).

So far, there have been many studies on the identification and functional analysis of SRS gene family in *Arabidopsis* (Greb et al., 2003), followed by studies on maize (He et al., 2020). By contrast, the SRS gene has yet to be reported in quinoa (*Chenopodium quinoa Willd.*), which has more nutritional value than any traditional food crop. Besides, quinoa is suitable for growing in high altitude areas (>3,500 m above sea level), and it is resistant to multiple abiotic stresses, including cold-tolerant, drought-tolerant, salt-tolerant and barren-tolerant. It has the potential to provide a highly nutritious food source that can be grown on marginal lands not currently suitable for other major crops (rice and maize). It is regarded as a facultative halophyte and shows a strong resistance to drought and low temperature as well. The nutritional value is as protein-rich as beef, and quality is as good as meat and milk proteins. However, despite its agronomic potential, quinoa is still an underutilized crop, with relatively few active breeding programs. Breeding efforts to improve the crop for important agronomic traits are needed to expand quinoa production worldwide. Currently, the lack of breeding for specific environments, the high photoperiodic sensitivity and the relatively low yield are the major factors that limit quinoa cultivation in nonnative areas. SRS transcription factors control a diverse range of developmental processes in plant, including root formation, leaf development, floral induction and flower development, and photomorphogenesis and the recent publication of quinoa genome provides an opportunity to identify the SRS genes of quinoa (Jarvis et al., 2017). Therefore, in this study, we identified ten SRS genes in quinoa, and systematically analyzed it from the basic physical and chemical properties, phylogeny, gene duplication, tissue expression, protein interaction and other aspects of the members of the gene family. These results provide a reference for further study on the function of SRS genes in quinoa, and provide a certain theoretical basis in breeding of quinoa.

## 2 Materials and methods

### 2.1 Search and identification of short internodes related sequence gene members of quinoa

The quinoa genome database (*Chenopodium quinoa* v1.0), including coding sequences, protein sequences and other information were downloaded from Phytozome v12 (https://phytozome.jgi.doe.gov/pz/portal.html). The amino acid sequences of the *Arabidopsis* SRS family members were downloaded from the *Arabidopsis* Information Resource (TAIR) (http://www.arabidopsis.org) (Poole, 2017) database, and SRS genes in quinoa were obtained by using their amino acid sequences for homologous alignment and removing redundant sequences. This screening was then combined with the SRS domain. Prediction of protein conserved domains using PFAM (http://pfam.xfam.org/family) (Finn et al., 2014), NCBI-CDD (https://www.ncbi.nlm.nih.gov/cdd/) (Marchler-Bauer et al., 2011) and SMART (http://smart.embl-heidelberg.de) (Schultz et al., 2000).

### 2.2 Basic physical and chemical properties of proteins and phylogenetic analysis

The basic physical and chemical properties of SRS proteins in quinoa were analyzed by ExPASy (https://web.expasy.org/protparam/) (Gasteiger et al., 1999), and the subcellular localizations of the SRS proteins were predicted by the Psort-Prediction (http://psort1.hgc.jp/form.html) (Gardy et al., 2005).

Phylogenetic trees of SRS family proteins of *Arabidopsis*, maize, tomato, spinach, *Nicotiana sylvestris*, *Selaginella moellendorffii*, *Physcomitrella patens* and quinoa were constructed by using Clustal W version 2.1 (Larkin et al., 2007) in MEGA7 (Kumar et al., 2016). ZmSRS, SoSRS, SlSRS, SmSRS, PpSRS, NsSRS genes come from PlantTFDB v5.0 (Tian et al., 2019). A phylogenetic tree was constructed using the maximum likelihood method, Poisson mode was used, repetition number was 1,000, other parameters are default. Evolutionary tree beautification through Evolview (https://evolgenius.info//evolview-v2/#login) (Zhang et al., 2012).

### 2.3 Gene structure and conserved motifs analysis

Based on the GFF annotation of the quinoa genome, the gene structure of the exon/intron of SRS genes was constructed by using the Gene Structure Display Server (GSDS) (http://gsds.cbi.pku.edu.cn/). Multiple Em for Motif Elicitation (MEME) program (http://meme-suite.org/tools/meme) (Steven, 1996) was used to analyze the conserved protein motifs. The number of motif searches was set as 10, and other parameters were default.

### 2.4 Chromosomal location and gene duplication analysis

The annotation information of the SRS genes in the quinoa database was used to determine the chromosomal location of members of the family. Fragment duplication pairs are detected on the plant genome duplication database server (http://www.plantgdb.org/). The amino acid sequence of partially duplicated CqSRS genes was predicted by Clustalw software. DnaSP v5.0 software (Librado and Rozas, 2009) was used to estimate of synonymous (Ks) and non-synonymous (Ka) replacement rate (Suyama et al., 2006), using the following formula to determine CqSRS gene duplication of time (millions of years ago, MYA) and divergence of time: T = Ks/2λ(*λ* = 6.56E-9) (Lynch and Conery, 2003).

### 2.5 Cis-acting element analysis and construction of protein interaction network

According to the quinoa genome database, 2000 bp DNA sequences upstream of the transcriptional initiation site of SRS family gene were extracted by TBtools (Chen et al., 2020), which was used as the promoter regions of regulation, the cis-regulatory elements of the promoter region of the SRS genes were retrieved and analyzed using the PlantCARE (http://bioinformatics.psb.ugent.be/webtools/plantcare/html/) (Lescot et al., 2002). Based on SRS proteins of *Arabidopsis thaliana*. The protein-protein interaction network of quinoa was further predicted by STRING software (https://string-db.org/) (Szklarczyk et al., 2015).

### 2.6 Secondary structure analysis and tertiary model prediction

The secondary structure of SRS proteins was analyzed by NPS@: GOR4 (https://npsa-prabi.ibcp.fr/cgi-bin/npsa_automat.pl?page=/NPSA/npsa_gor4.html) (Combet et al., 2000). Meanwhile, we predicted the tertiary structure of the protein by the swiss-model server (Kelley et al., 2015).

### 2.7 Plant materials and treatments

L-2 (Longli No.2 from Gansu Academy of Agricultural Sciences) was used as material. It was identified by Yang (Yang, 2016) and in February 2016 through the Gansu Province crop variety examination and approval committee (2,016,004). The growth period is 152–160 days and the plant height is 196.2–243.5 cm. The grains are white, round and flaky, with a diameter is 1.6–2.4 mm and a 1,000-seed weight is 2.9–3.3 g. The grains contained crude protein (dry base) is 165.10 g/kg, crude fat (dry base) is 52.00 g/kg, crude ash (dry base) is 34.17 g/kg, lysine (dry base) is 7.00 g/kg and total phosphorus (dry base) is 5.62 g/kg. The quinoa was disinfected in 10% sodium hypochlorite for 20 min, then rinsed with sterile water for 5 times, and seeded on MS solid medium. It was cultured in a greenhouse at 24 ± 1°C for 14/10 h in light/dark light cycle until germination. The germinated seeds were planted in a 1:1:1 tray containing sand, perlite and peat, and cultured in the growth chamber (relative humidity 60–70%, illumination time 12 h, day-night temperature 28°C/18°C). After the seedlings had grown for about 2 months, they were placed in an incubator at 4°C for low-temperature treatment. Under salt stress, 100 mmol/L NaCl was sprayed on the surface of plant leaves. In ABA treatment, 200uM ABA was sprayed on the surface of plant leaves. CK was the plant under normal growth conditions. The root was collected at 0, 2, 4, 8, and 12 h after treatment. Under drought stress, stop watering at the beginning of treatment and collect the leaves and roots of quinoa at 0, 3, 5 and 7 days after treatment. And three biological replicates were conducted at each time point. The collected leaves and roots were temporarily stored in liquid nitrogen, and then stored at −80°C for the subsequent quantitative test.

### 2.8 Expression analysis of short internodes related sequence genes, ribonucleic acid extraction and real-time quantitative polymerase chain reaction (Quantitative reverse transcription-polymerase chain reaction)

The SRS gene expression data of quinoa were obtained from transcriptomic data for the different tissues and organs of quinoa (No.: PRJNA394651) and the aboveground tissues of quinoa seedlings under drought, high temperature, salt and low phosphorus stress (No.: PRJNA306026). RNA-sequencing (RNA-seq) data (PRJNA394651 and PRJNA306026) were downloaded from the National Center for Biotechnology Information (NCBI, https://www.ncbi.nlm.nih.gov/) (Zou et al., 2017). The log2 method was used to de-standardize the data.

Total RNA was extracted from each sample using the Trizol total RNA extraction kit (Sangon, Shanghai, China, SK1321), and cDNA was obtained using the Superscript™III reverse transcriptase kit (Invitrogen). qRT-PCR primers were designed using Premier 5 (Livak and Schmittgen, 2001). And normalized with Elongation Factor 1 alpha (*EF1α*, Supplementary Table S6). The concentration and purity of RNA and cDNA extracted were determined by a quantitative ultraviolet Spectrophotometer Q5000 (UV-VIS), and q-RT-PCR analysis was done with 2× lyect-SYbr-green-Pcr-mix (Qiagen) in the real-time PCR system of American Applied Biosystems, the program is shown as follows: Denaturation at 95°C for 3 min, followed by denaturation at 95°C for 10 s for 40 cycles, and finally annealing/extension at 60°C for 1 min (Zhang S. et al., 2013). Relative gene expression level was calculated using the 2^−∆∆Ct^ method (Livak and Schmittgen, 2001). Each experiment was repeated in triplicate using independent RNA samples.

### 2.9 Statistical analysis

Data quantified from the qRT-PCR of the three biological replicates were analyzed with two-way ANOVA using SPSS (version 19) and statistically evaluated using the Duncan method. A difference was considered statistically significant level of *p* < 0.05.

## 3 Result

### 3.1 Basic physical and chemical properties

Finally, a total of 10 SRS genes in quinoa were identified, and named *SRS01*-*SRS10*. The coding sequence (CDSs) of the members of this family is between 432–1,113 nucleotides, and the coding amino acid varying from 143 to 370 aa in length (Table 1), with an average of 244 aa. Except for CqSRS01 and CqSRS02, the pI of the coding proteins is less than 7, and the hydrophobicity index of CqSRS proteins is less than 0, indicating that these proteins are hydrophilic. Subcellular localization predictions showed five CqSRS genes were localized in the cytoplasm, and a few were localized in the nucleus, plasma membrane and mitochondria. The structure and stability of CqSRS proteins are determined by the instability index, which provided an estimate of protein stability. In this study, six CqSRS proteins were unstable, with the instability index greater than 40. Four CqSRS proteins may be stable, with an index between 32.45 and 39.45.11.

**TABLE 1:** Characteristics of short internodes related sequence genes in quinoa.

Gene accession no	Gene	Size (aa)	Molecular weight (D)	Isoelectric point	Instability index	GRAVY	Subcellular localization
AUR62000185-RA	*CqSRS01*	370	37,758.52	8.33	36.33	−0.452	plasma membrane
AUR62006536-RA	*CqSRS02*	241	24,289.62	8.90	39.45	−0.487	nucleus
AUR62007206-RA	*CqSRS03*	312	35,034.26	6.80	52.91	−0.854	mitochondrion
AUR62007636-RA	*CqSRS04*	244	26,367.17	5.56	45.09	−0.491	cytoplasm
AUR62007664-RA	*CqSRS05*	246	26,717.52	5.45	47.68	−0.591	cytoplasm
AUR62010428-RA	*CqSRS06*	143	15,336.89	5.92	52.51	−0.290	cytoplasm
AUR62014445-RA	*CqSRS07*	170	17,896.76	6.08	40.35	−0.449	mitochondrion
AUR62016794-RA	*CqSRS08*	246	26,764.40	4.81	32.45	−0.539	cytoplasm
AUR62018795-RA	*CqSRS09*	312	34,969.13	5.85	51.62	−0.799	mitochondrion
AUR62034552-RA	*CqSRS10*	163	16,857.59	5.60	37.67	−0.374	cytoplasm

Note: GRAVY, represents Grand average of hydropathicity.

### 3.2 Evolutionary relationships and classification of short internodes related sequence genes

**FIGURE 1:**
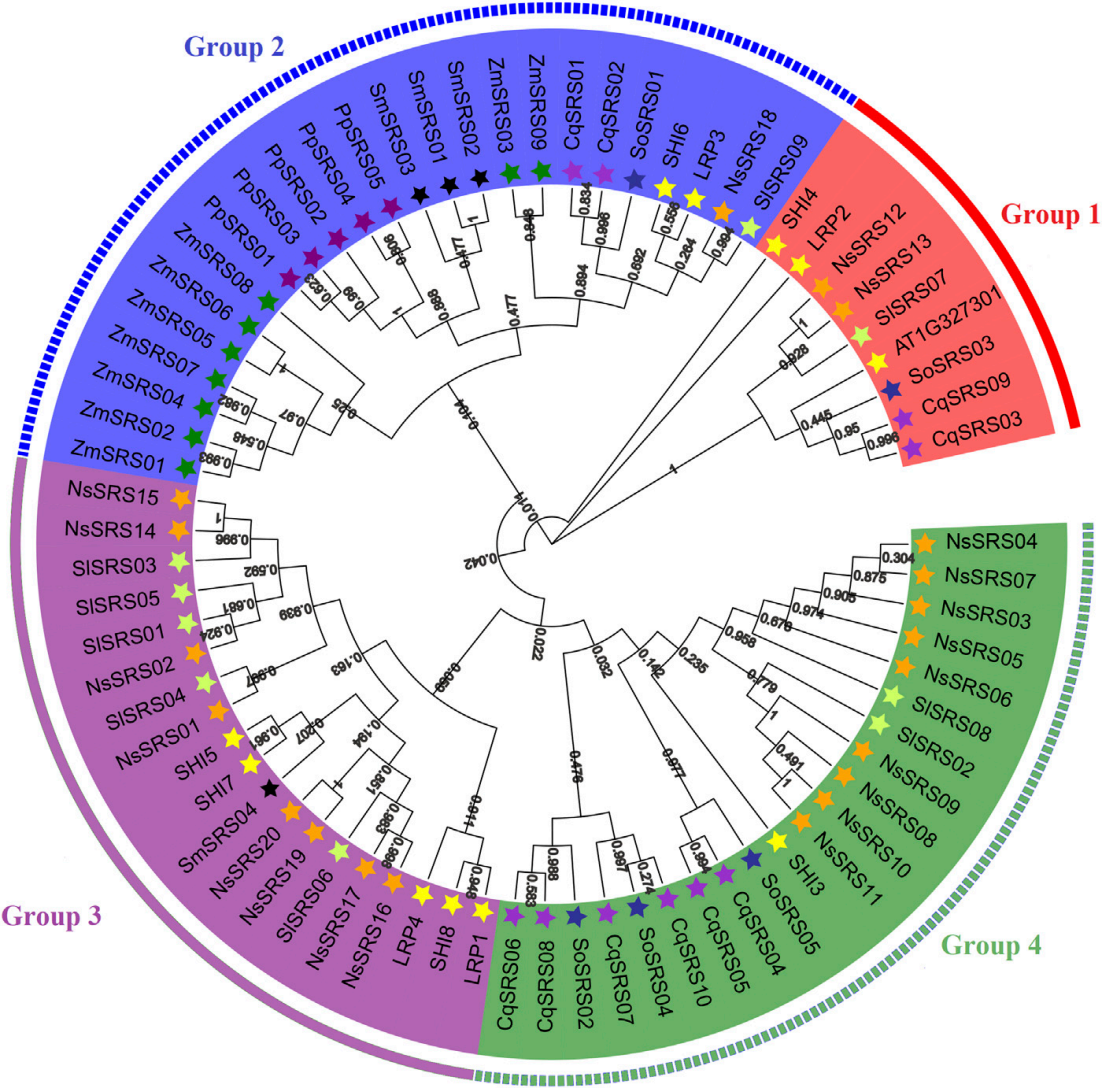
Phylogenetic relationships of SRS proteins from *Arabidopsis*, *Zea mays L*, *Solanum lycopersicum*, *Spinacia oleracea L, Nicotiana sylvestris, Selaginella moellendorffii*, *Physcomitrella patens* and quinoa. The proteins clustered into four subgroups, denoted with different colors to represent subfamilies as follows: Group1 (red), Group 2 (blue), Group 3 (purple), Group 4 (green). The information of the SRS family members from *Arabidopsis, Zea mays L, Solanum lycopersicum, Spinacia oleracea L, Nicotiana sylvestris, Selaginella moellendorffii, Physcomitrella patens and quinoa* was listed in the supporting information (Supplementary Table S1). A phylogenetic tree was constructed using the maximum likelihood method, bootstrap values based on 1,000 replications were calculated.

To study phylogenetic relationships between the SRS proteins of quinoa, we constructed phylogenetic trees from 73 protein sequences of *Arabidopsis* (11), maize (9), tomato (9), spinach (5), *Nicotiana sylvestris* (20), *Selaginella moellendorffii* (4), *Physcomitrella patens* (5) and quinoa (10). According to the topological structure of the tree, all plants share a common ancestor with the SRS genes. Meanwhile, according to their homology, these genes are divided into 4 subfamilies (Figure 1 and Supplementary Table S1). The first group has 9 SRS genes, the second group contains 24 genes, the third group contains 19 genes and the fourth group contains 21 genes. At the same time, we can observe that there are four pairs of orthologous genes in these 8 species (*SlSRS09/NsSRS18, SlSRS01/NsSRS02, SlSRS04/NsSRS01, SoSRS04/CqSRS10*), and 21 pairs of paracentric homologous gene pairs (four pairs in quinoa: *CqSRS03/CqSRS09*, *CqSRS01/CqSRS02, CqSRS04/CqSRS05, CqSRS06/CqSRS08*). There are one pair of homologous genes between quinoa and spinach*,* indicating that there is no obvious difference between these two species in the evolutionary process. In addition, we found that there was no clear division between the SRS genes of moss, pteridophyte, gymnosperm, monocotyledon, and dicotyledon, suggesting that they may have come from the same ancestor.

### 3.3 Chromosomal location and gene duplication analysis

**FIGURE 2:**
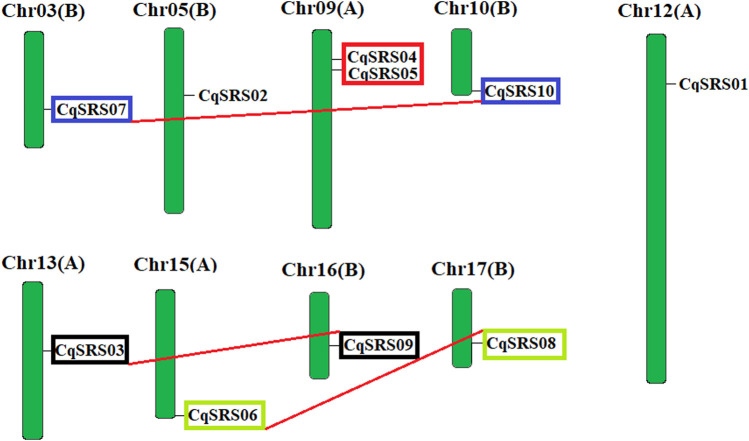
Chromosome mapping of CqSRS genes in quinoa.

To verify the relationship between genetic differentiation and gene duplication, we identified the chromosomal locations of CqSRS genes (Figure 2). In this study, the chromosomal locations of CqSRS gene family members were obtained through the quinoa genome (David et al., 2017). Ultimately, 10 CqSRS genes were located on the 9 chromosomes of quinoa, chromosomes 3, 5, 10, 12, 13, 15, 16, 17 each contain one SRS gene, chromosome 9 contain two SRS genes.

The duplication of CqSRS genes was further tested. Previous studies showed that five or fewer genes localization within the range of 100 kb on the same chromosome are usually considered as tandem duplication (McGregor et al., 2017). Therefore, there is no tandem duplication in this study. We based on two conditions (comparison rate of two genes >75%, comparing similarity >75%) to screen for duplicated genes, and finally identified to 4 pairs of duplicated genes (Table 2), and they respectively location on different chromosomes, thus belongs to the duplicated gene fragments, and duplication occur between 6.830 and 14.151 MYA. The history of the selection acting on the coding sequence can be measured in terms of the ratio of non-synonymous substitutions to synonymous substitutions (Ka/Ks). Ka/Ks < 1 was selected for purification. When the two sequences drift in neutral and special, Ka/Ks = 1. At specific sites of positive selection, Ka/Ks > 1. Ka/Ks values of four gene pairs in this study were all less than 1, indicating that the evolution of all gene pairs was mainly influenced by purification selection, and purification selection could inhibit the differentiation of duplicate genes.

**TABLE 2:** Gene duplication in CqSRS family in quinoa.

Duplicated SRS gene1	Duplicated SRS gene2	Ka	Ks	Ka/Ks	Date (MYA)T = Ks/2λ	Selective pressure	Duplicate type
CqSRS03	CqSRS09	0.021	0.116	0.180	6.830	Purifying selection	Segmental
CqSRS04	CqSRS05	0.049	0.185	0.263	10.959	Purifying selection	Segmental
CqSRS06	CqSRS08	0.174	0.239	0.728	14.151	Purifying selection	Segmental
CqSRS07	CqSRS10	0.011	0.116	0.098	6.836	Purifying selection	Segmental

Note: The non-synonymous (Ka) and synonymous substitution rate (Ks); millions of years ago (MYA).

### 3.4 Analysis of gene structure and conserved motifs

**FIGURE 3:**
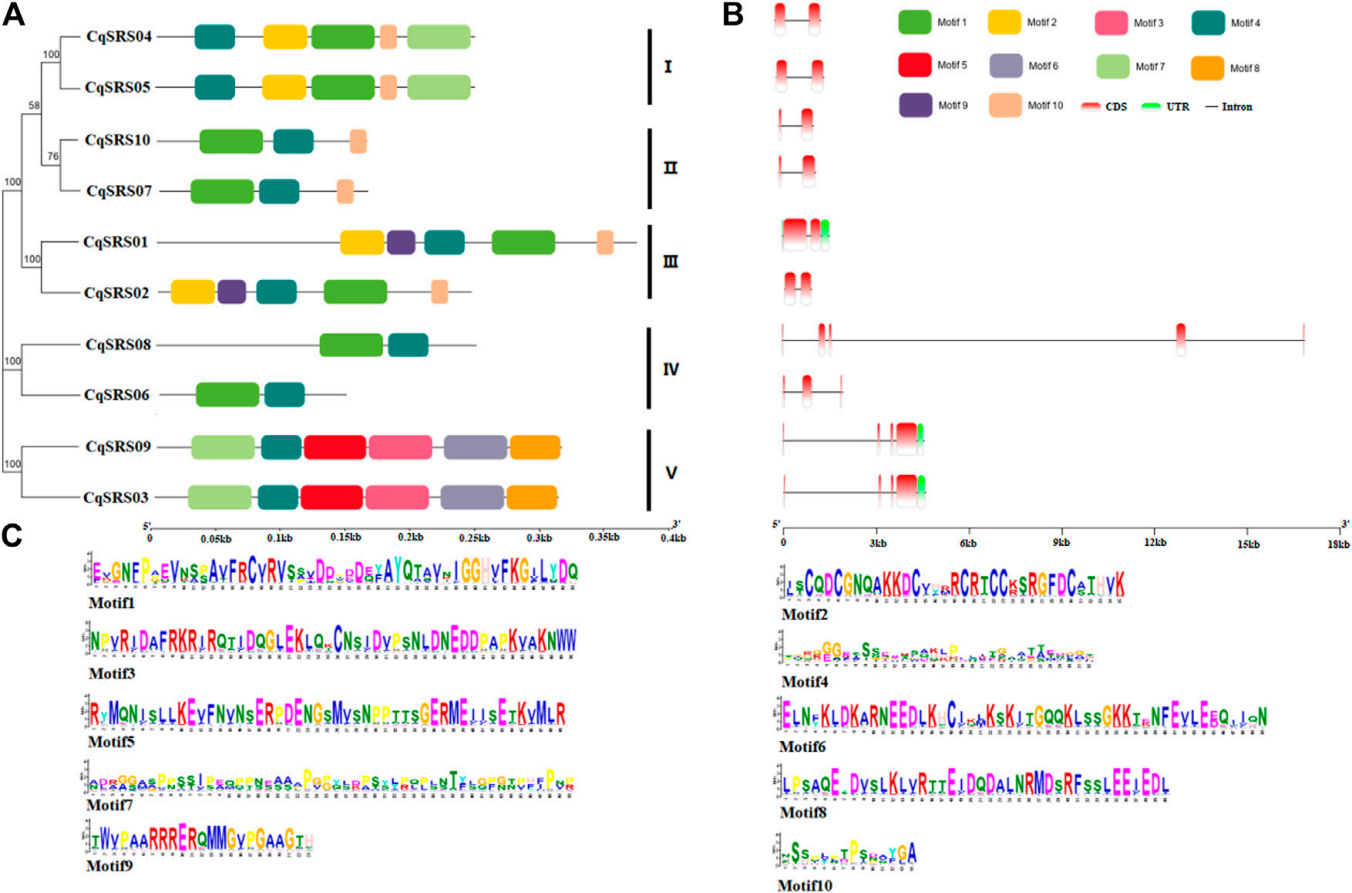
Structural analysis of CqSRS genes in quinoa. An unrooted phylogenetic tree was constructed based on the full-length sequences of CqSRS proteins using the N-J method in MEGA7. Bootstrap values based on 1,000 replications were calculated. **(A)** The distribution of motifs in SRS proteins. **(B)** The exon-intron structure of the SRS genes. **(C)** The amino acid composition of each motif, motif sequences in Supplementary Table S2.

On the one hand, the diversity of gene structure reflects the evolutionary relationship of gene families. Meanwhile, the intron-exon pattern plays a key role in gene function. Therefore, we analyzed the exon/intron pattern of members of this family by comparing the coding sequence with the corresponding genomic DNA sequence. Results showed that the number of exons CqSRS between 2 and 5, and same subfamily genes have a similar introns/exon mode. For example, the number and length of exons of corresponding genes in subfamilies 1, 2 and 3 are highly similar, and the genes are highly homologous to each other, suggesting that they are in the process of evolution was derived from a common ancestor, or maybe the result of a genetic duplication (Figure 3). The conservative motifs of CqSRS proteins were analyzed by using MEME and 10 conserved motifs were identified. It was found that motif 4 exists in all CqSRS genes, motif 1 exists in most CqSRS genes, and motif 3, 5, 6 and 8 only exist in CqSRS03 and CqSRS09. Motif 9 may be the basis for the division of CqSRS01 and CqSRS02 in the same branch. Most CqSRS genes with similar gene structure have the same motif compositions and similar functions.

### 3.5 Cis-acting element analysis and construction of protein interaction network

**FIGURE 4:**
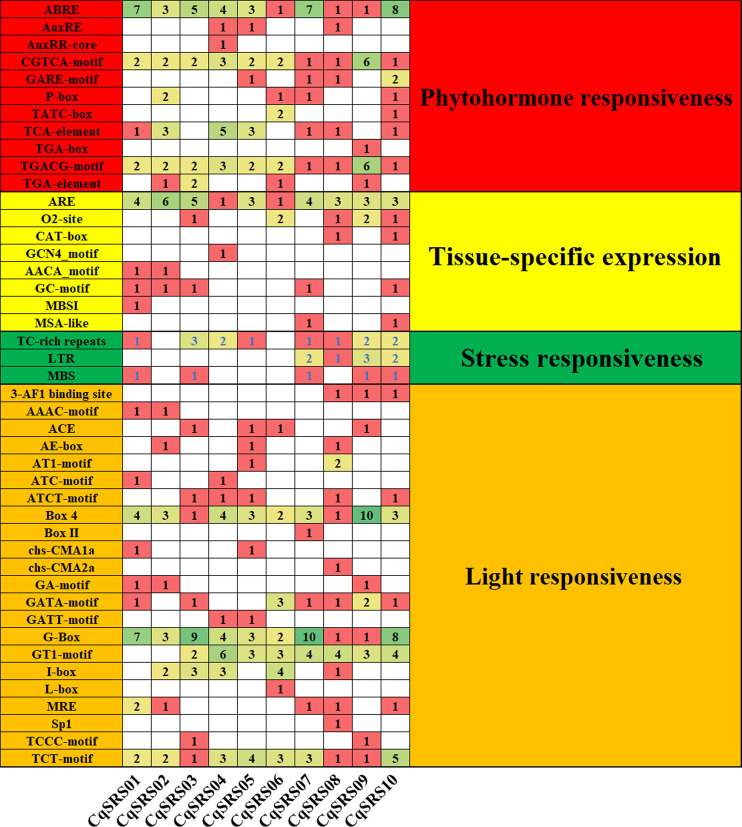
Cis-acting components of quinoa SRS genes. All promoter sequences (2000 bp) were analyzed. Cis-acting element names and functions can be found in Supplementary Table S3.

In order to study the cis-acting elements in the CqSRS genes promoter regions, the promoter sequences of CqSRS genes were analyzed by PlantCARE. We found that all CqSRS genes promoter regions contained one or more TATA-box. Meanwhile, we found a total of 44 elements related to plant hormone response elements, light response elements, stress response elements and tissue-specific expression elements in the upstream region of the promoter (Figure 4 and Supplementary Table S3). The light response element was the most cis-acting element, followed by plant hormone and stress response element, and the tissue-specific expression element was the least. Plant hormones such as auxin, abscisic acid, gibberellin and jasmonic acid play a key role in plant resistance to adversity. In this study, CqSRS genes contained a variety of hormone-related elements. ABRE, CGTCA-motif, TGACG-motif and other plant hormone elements existed in all CqSRS genes in the form of a single copy or multiple copies. Some genes (*CqSRS02*, *CqSRS05*, *CqSRS07*, *CqSRS06*, CqSRS07, *CqSRS08* and *CqSRS10*) contained five hormone response elements, including abscisic acid (ABRE), AuxRE (AuxRE, AUXRR-core, CGTCA-motif and TGA-Box), salicylic acid (TCA-element), gibberellin (GARE, P-box and TATC-Box) and methyl jasmonate (TGACG-motif). CqSRS genes also contain some tissue-specific elements, including meristem expression elements (CAT-box) and endosperm expression elements (GCN4_motif and AACA-motif). In addition, the family also contain a small number of stress response elements, including low-temperature response elements (LTR), drought induction elements (MBS), and defense and stress response elements (TC-rich repeats).

**FIGURE 5:**
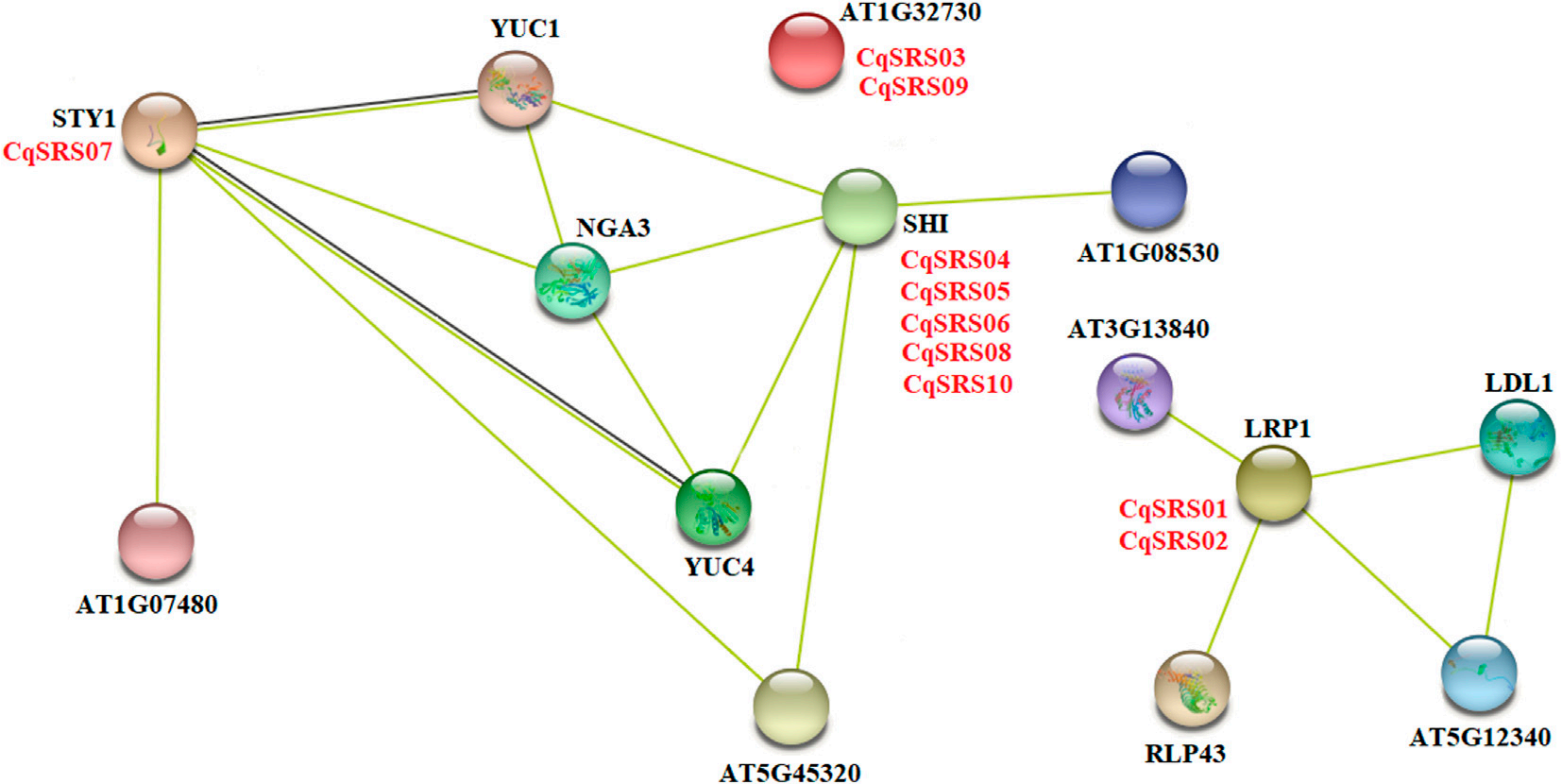
The potential interaction network of CqSRS based on the *Arabidopsis* and quinoa.

To further investigate which protein interact with SRS family members, we researched Arabidopsis proteins homologous to quinoa proteins appear in the Arabidopsis network, which indicates that similar protein-protein interactions may occur in quinoa. As can be seen from the figure below, 10 CqSRS proteins appear in the known *Arabidopsis* protein interaction network (Figure 5). Among them, the protein sequence of *AtSTY1* is highly similar to that of CqSRS07, AtSTY1 gene, as a transcriptional activator, can bind to the DNA on 5' -ACTCTAC 3′ and promote the expression of auxin homeostasis regulation genes (such as *YUC* gene), as well as genes affecting stamen development, cell amplification and flowering time, so *CqSRS07* gene may have a similar function (Spyropoulou et al., 2014). *AtLRP1* gene has been identified as an auxin-induced gene, and its expression was regulated by histone deacetylation, so the expression of *CqSRS01* and *CqSRS02* may also be regulated by auxin signal (Sohlberg et al., 2006). Five CqSRS genes (*CqSRS04*, *CqSRS05*, *CqSRS06*, *CqSRS08* and *CqSRS10*) are similar to *AtSHI* gene, revealing their synergistic effect with other related proteins (*NGA3* and *YUC1*) to regulate pistillate, stamen and leaf development in a dose-dependent manner and control apical basal configuration, and promote pistil development and stigma formation, and affect the development of blood vessels during pistil development (Islam et al., 2013).

### 3.6 Secondary structure analysis and tertiary model prediction

**FIGURE 6:**
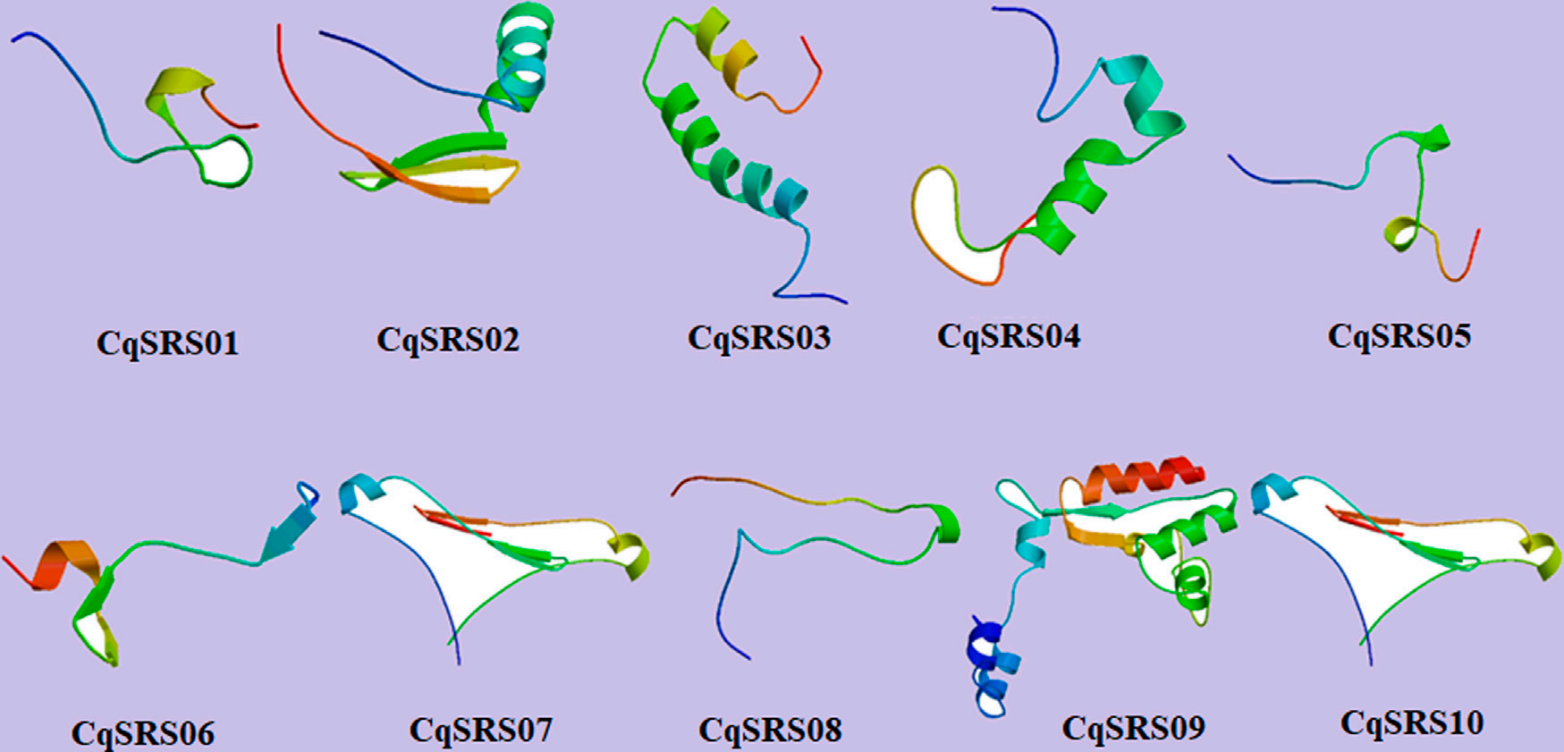
Tertiary Structure Prediction of SRS genes in quinoa.

In order to better understand the structural characteristics of CqSRS proteins, a third-level model of the protein family was predicted using swiss-model, and the results showed that members in the same subgroup had similar third-level structures (Figure 6). The secondary structure consists of random coil (Cc), extended strand (Ee), and alpha helix (Hh), of which random coil account for the largest proportion (more than 50%) (Supplementary Table S4).

### 3.7 Ribonucleic acid-seq analysis

**FIGURE 7:**
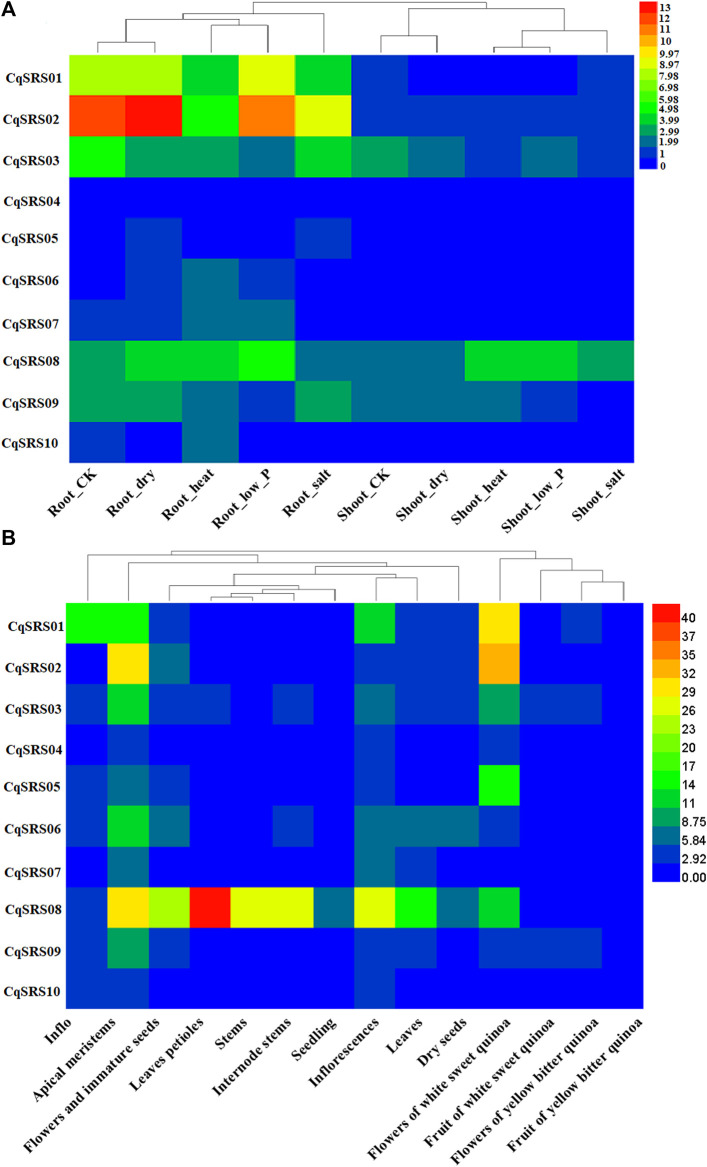
The expression profiles of SRS genes in different treatments, developmental stages and tissues of quinoa. **(A)** CqSRS expression patterns under different treatments, including Root-CK, Root-dry, Root-heat, Root-low_P Root-salt, Shoot-CK, Shoot-dry, Shoot-heat, Shoot-low_P and Shoot-salt. **(B)** CqSRS expression patterns under different developmental stages and tissues, Inflo, Apical meristems, Flowers and immature seeds, Leaves petioles, Stems, Internode stems, Seedling, Inflorescences, Leaves Dry, seeds, Flowers of white sweet quinoa, Fruit of white sweet quinoa, Flowers of yellow bitter quinoa, Fruit of yellow bitter quinoa. Gene expression was calculated by FPKM. RNA-sequencing (RNA-seq) data (PRJNA394651 and PRJNA306026) were downloaded from NCBI. We standardized the data using the Log2 method.

We used transcriptome data to study the expression patterns of genes in this family. The results of heatmap showed that most CqSRS genes showed a low expression under different treatments (Figure 7 and Supplementary Table S5). For example, *CqSRS04*-*CqSRS06* and *CqSRS10*. *CqSRS01*-*CqSRS03* genes are highly expressed in roots under high temperature, low phosphorus, drought and salt stress, and these genes may play a key role under abiotic stress. In addition, the expression of CqSRS genes in tissues and organs at different development stages of quinoa was also significantly different. Almost all the genes high expression in apical meristems and flowers of white sweet quinoa. Most genes (except *CqSRS08*) are low expression in leaves. The expression pattern of *CqSRS08* was different from that of other proteins. The expression of *CqSRS08* was high in all tissues, especially in leaves up to 43 times, indicating that some SRS genes have the characteristics of tissue expression.

### 3.8 Expression profiling of CqSRS genes in different treatments

**FIGURE 8:**
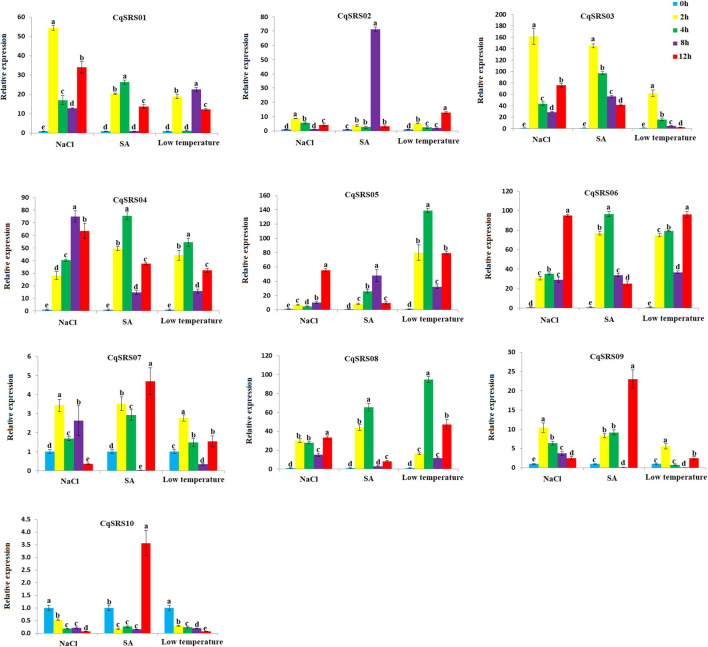
Expression profiles of 10 SRS genes using qRT-PCR analysis in quinoa. Values represented the mean ± standard error of the mean (SEM) of three biological replicates with three technical replicates at different treatments. Error bars indicated the SEM among the three experiments. Different lowercase letters represent significant levels of difference (*p* < 0.05).

Stress seriously affects the growth and development of plants, so qRT-PCR was used to analyse the expression patterns of the family members in roots under stress (Figure 8). The results showed that all SRS genes were responsive to SA, NaCl and low-temperature. The expression levels of different CqSRS genes were significantly different under different stress. In SA treatment, some genes (*CqSRS02*, *CqSRS03*, *CqSRS05* and *CqSRS06*) showed the same pattern of first increasing and then decreasing, and some genes (*CqSRS01*, *CqSRS04*, CqSRS07-*CqSRS10*) showed the lowest expression after 8 h treatment. Under NaCl and low-temperature treatment, most of the genes had the same expression pattern (2 or 12 h expression level was extremely significant), and the expression level of the treatment was significantly higher than that of the control group. However, the expression of *CqSRS10* gene in NaCl and low temperature was lower than that in control. These results showed that the CqSRS gene family members in most roots were strongly induced by 100 mmol/L NaCl, 200 umol/L ABA and 4°Cunder different treatments, and only a few members were not sensitive to abiotic treatment.

**FIGURE 9:**
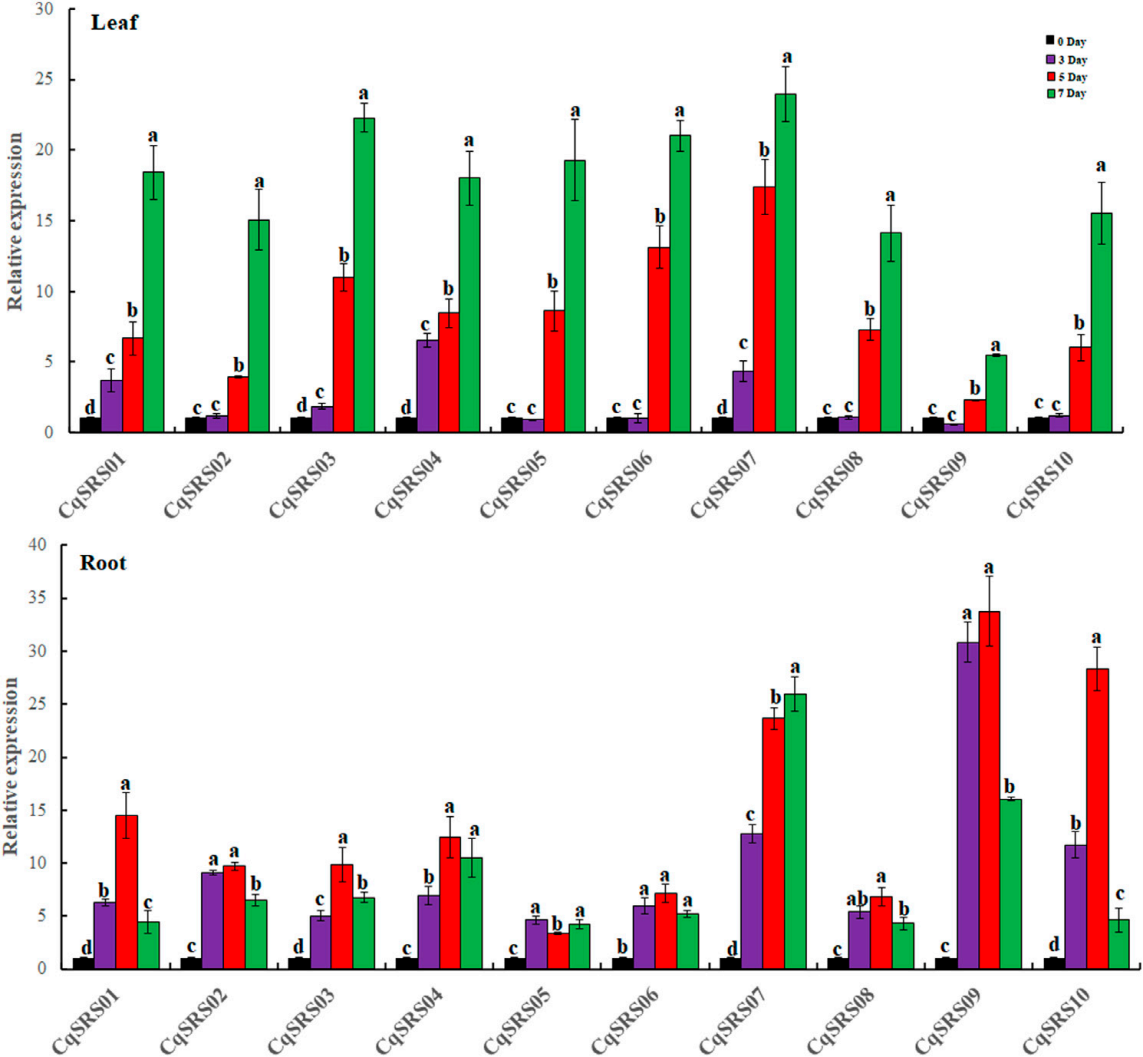
RT-qPCR analysis of the 10 CqSRS genes under drought in leaf and root. Values represented the mean ± standard error of the mean (SEM) of three biological replicates with three technical replicates at different treatments. Error bars indicated the SEM among the three experiments. Different lowercase letters represent significant levels of difference (*p* < 0.05).

In addition, we studied the expression patterns of the SRS genes under drought stress by qRT-PCR (Figure 9). It was observed that all members of the SRS family were responsive to drought stress in leaves, and the expression pattern of 10 SRS genes increased with the extension of drought stress time, and reached the maximum on the 7th day after treatment. The expression of seven genes (except *CqSRS01*, *CqSRS04* and *CqSRS07*) had no significant difference between the control group and the control group at the 3rd day, indicating that drought had little effect on these genes within 0–3 days, and the expression of 10 genes increased significantly within 3–7 days, the results indicated that drought stress induced the expression of SRS genes in leaves at this stage, which could respond to drought stress. Different expression patterns of SRS genes were observed in the roots. The expression of eight genes (except CqSRS05 and CqSRS07) increased first and then decreased, and reached the maximum on the 5th day after treatment. Interestingly, we observed that *CqSRS09* gene responded strongly to drought stress in roots but least in leaves, suggesting that *CqSRS09* may play a major role in quinoa roots.

## 4 Discussion

Plants encounter various biotic and abiotic stresses during their growth and development, and transcription factors play an important role in a series of biological processes throughout the life cycle of plants. As an important transcription factor, SRS is significantly characterized by a conservative ring-finger zinc finger domain at the N-terminal of the protein, many SRS genes take part in apical gynoecium development and mediate stigma development (Gomariz-Fernández et al., 2017). Meanwhile, research showed SRS genes are correlated with crop yields (Kuusk et al., 2006). However, the identification of the SRS genes has been reported in maize, rice and *Arabidopsis thaliana* (Kuusk et al., 2006; Yang et al., 2020; Yang et al., 2021; Büyük et al., 2022). It has not been reported in quinoa. Quinoa is a highly resistant crop, and its genome sequencing can help us to identify the resistance genes and improve the genetic improvement of the crops. Therefore, in this study, we identified 10 SRS genes from quinoa, which was consistent with the results of *Arabidopsis thaliana* (11 SRS) (He et al., 2020) and maize (11 SRS) (Kuusk et al., 2006), indicating that the number of the SRS gene family in different species was not significantly different, and it also reflects that the three species did not undergo large-scale genome-wide replication during the course of evolution. Moreover, the number of amino acids, isoelectric point and molecular weight of the family members’ proteins were significantly different, which may be due to the different functions of the family members during their growth and development. The distribution of SRS genes in maize, *Arabidopsis*, tomato, spinach and quinoa may be the result of gene differential amplification after the differentiation of monocotyledons and dicotyledons from the same ancestor. At the same time, the number of SRS genes in the eight species was relatively small, indicating that the retention and duplication of genes in different species were basically consistent with similar evolutionary constraints (Airoldi and Davies, 2012). 10 SRS genes were distributed on 9 chromosomes, and no tandem duplication was found, which was consistent with the study on SRS family genes in maize (Kuusk et al., 2006).

In order to understand the structural diversity of SRS genes in quinoa, the gene structure was analyzed. Previous research has shown that an intron-rich gene can lose multiple introns simultaneously, resulting in an intron-free genetic ancestor, and the intron-free genes in eukaryotic genomes may be derived from the horizontal gene transfer of ancient prokaryotes (Wang et al., 2019). The different splicing states of exons and introns may be meaningful to the evolution of CqSRS genes. In this study, the number of introns in subgroups 1, 2 and 3 were small and similar, which may be due to intron loss during the evolution of the SRS genes, subgroups 4 and 5 have similar intron numbers. Although introns have no effect on protein sequence, their relative positions provide clues to predict how genes and their corresponding proteins evolve and further promote the structural diversity of genes (Rogozin et al., 2000). This diversity of gene structure may drive the evolution of gene families, and may enable genes to have new functions that can help plants better adapt to environmental changes (Fan et al., 2014). Meanwhile, we identify 10 conserved motifs and the CqSRS genes in the same subfamily were found to have the same motif composition, which indicates that the genes of the same subfamily have similar functions. Although the 10 SRS genes share a common conserved motif 4, they also have their unique conserved motifs, and different motif composition may contribute to the functional diversity of CqSRS members (Liu and Chu, 2015). The study on the SRS genes structure and conserved motif of quinoa provided a reference for further study on the evolution of the SRS family of quinoa.

Recent studies have shown that gene duplication not only is important in the expansion and rearrangement of genomes in the evolutionary process, but also induces the diversification of gene functions (Zhang X. et al., 2013). The three most important evolutionary patterns are fragment duplication, tandem duplication, and transposition events (Mao et al., 2016). Five or fewer genes located within the 100 kb range of a chromosome are generally considered tandem duplication, while gene duplication occurring on different chromosomes is considered fragment duplication (Liu et al., 2011; Hu et al., 2015). In this study, there are four pairs of gene duplication, and four pairs of genes belong to fragment duplication, indicating that segmental duplication mainly contribute to the evolution of CqSRS genes in quinoa. In addition, Ka/Ks of these four pairs of genes were all less than 1, indicating that purification selection plays a major role in the expansion of SRS genes in quinoa, which is consistent with previous studies (Cao et al., 2019). Meanwhile, these duplicated genes may have retained ancestral functions during evolution.

Cis-acting elements are important in plant defense against various biotic and abiotic stresses (Zhao et al., 2016), and they can specifically bind with transcription factors to regulate gene transcription (Riechmann et al., 2000). In this study, we identified several cis-acting elements associated with auxin, gibberellin, salicylic acid (SA), abscisic acid (ABA), and methyl jasmonate (MeJA) in the promoter region of the CqSRS genes. These hormone response elements play a key role in various life activities in plants. ABRE (ABA response elements, 40), CGTCA-motif (MeJA response elements, 22), and TGACG-motif (MeJA response elements, 22) were found in all CqSRS genes, indicating that these elements are highly conserved in the CqSRS family. At the same time, virtually SRS genes contain two or more identical copies of the cis-acting elements. This may play a role in enhancing regulation of gene transcription and adapting to environmental changes. ARE is necessary for anaerobic induction and exists in multiple copies of all CqSRS genes. The analysis of CqSRS genes promoter region revealed the existence of various cis-acting elements, which regulated the expression level of genes.

The analysis of CqSRS tertiary structure and protein-protein interaction is helpful to further understand the function of CqSRS genes. In this study, genes in the same branch have similar protein structures, such as CqSRS07 and CqSRS10, so they may have similar functions. Furthermore, we constructed a network of protein interaction between *Arabidopsis* and quinoa. Previous studies have shown that *AtLRP1* gene has been identified as an auxin induced gene, and its expression is regulated by histone deacetylation, so the expression of CqSRS01 and CqSRS02 may also be regulated by auxin signaling (Singh et al., 2020). In *Arabidopsis thaliana*, *SHI* gene plays a role in the regulation of stamen development, cell amplification and flowering time (Veronika et al., 2012), so the five CqSRS genes that are highly similar may have similar functions.

Gene expression pattern is closely related to gene function. In this study, the expression levels of most genes were significantly increased under different treatments, indicating that most genes are co-expressesed under a variety of adverse conditions, this is consistent with Yang’s research (Yang et al., 2021), *CqSRS10* gene was up-regulated in leaves under SA stress, but down-regulated under salt and low temperature stress. Additionally, the same gene showed different expression patterns under different stress. Previous studies showed that SRS gene (LOC_Os01g72490) in maize could be induced by GA, but inhibited by PB, indicating that GA and PB activate antagonistic mechanism (Yang et al., 2020). It shows that SRS genes play an important role in plant development regulation and response to abiotic stress; they may be involved in the regulation of various responses related to stress and hormones. It was also found that the expression patterns of SRS genes in quinoa were different, indicating that these genes may participate in different biological processes or play different biological functions. Most genes are a low expression in the leaves; this is consistent with studies in *Arabidopsis* (Yang, 2016). Although some genes were homologous, their expression levels in roots and leaves under drought stress were quite different, indicating that some SRS genes showed tissue dependence (*CqSRS01* and *CqSRS08*). Meanwhile, some subfamilies have different gene expression patterns; it is speculated that the difference in the expression of different SRS between the same subfamily may be related to the sequence out of the conservative motif. Studies have shown that *OsSHI1* in rice is highly expressed in roots but not in leaves, and some AtSRS genes are highly expressed in flowers and roots but not in leaves (Kuusk et al., 2006). These results are consistent with our study.

## 5 Conclusion

Finally, a total of 10 SRS genes were identified in quinoa. Phylogenetic tree analysis showed that CqSRS genes are divided into three groups, and the gene structure showed that the number of exons of CqSRS was between 2–5. The gene expansion of this family may be the result of fragment duplication. Promoter analysis revealed that there are a total of 44 elements related to plant hormone response, light response, stress response, and tissue-specific expression. Transcriptome data analysis showed that CqSRS genes have different expression patterns, qRT-PCR indicated that all SRS family genes are responsive to SA, NaCl and low temperature. These results indicated that the main expression patterns and detailed functions of quinoa SRS genes are different in different developmental stages. Therefore, future research on these CqSRS genes may reveal the different functions of quinoa SRS genes. This study can further deepen our understanding of the molecular evolution and function of the quinoa SRS gene family, and provide a theoretical basis for further research on the SRS family in quinoa.

## Data Availability

The datasets presented in this study can be found in online repositories. The names of the repository/repositories and accession number(s) can be found in the article/Supplementary Material.
